# MXene/Bi_2_O_3_ Nanocomposites as Supercapacitors for Portable Electronic Devices

**DOI:** 10.1021/acs.energyfuels.5c04057

**Published:** 2025-10-27

**Authors:** Nanasaheb M. Shinde, Martin Pumera

**Affiliations:** Advanced Nanorobots & Multiscale Robotics Laboratory, Faculty of Electrical Engineering and Computer Science, 48278 VSB—Technical University of Ostrava, 17. listopadu 2172/15, 70800 Ostrava, Czech Republic

## Abstract

2D MXene nanosheets often encounter challenges such as aggregation and restacking, which can significantly decrease their performance in supercapacitor applications. Herein, we prepared the Bi_2_O_3_ nanoflowers decorated MXene electrode via the coprecipitation method. The insertion of Bi_2_O_3_ nanoflowers over the MXene nanosheet not only effectively resolves the restacking challenge of the MXene nanosheet but also improves the overall electrochemical performance of the MXene/Bi_2_O_3_ nanocomposite. The fabrication of an MXene/Bi_2_O_3_//MnCo_2_O_4_ supercapacitor device used for powering portable electronic devices confirmed that the lab-scale innovation could be turned into practical energy technologies. This work highlights a room-temperature synthesis strategy for the MXene-based nanocomposite, leading to high-performance, laboratory-scale prototype energy storage devices. Furthermore, this self-assembly process of MXene-metal oxide nanocomposites at room temperature opens new avenues for energy storage applications.

## Graphical abstract

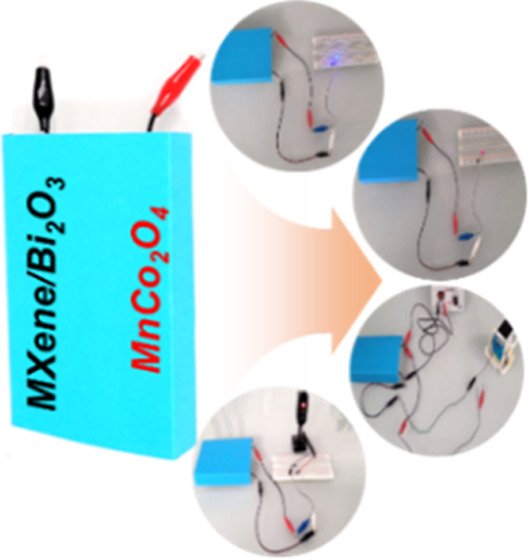

## Introduction

Supercapacitors have been broadly used as power-holding strategies as a result of their swift electrochemical response, excellent stability, moderate power density, higher energy density, and long cycling life.^1,2^ From the electrochemical data analysis, it was found that supercapacitors are located in between capacitors and batteries.^3–6^ The electrochemical performance of the supercapacitor was dependent on the fabricated base electrode material.^7–12^ 2D MXene is a promising material, including constituent elements as transition-metal carbides.^13,14^ MXene shows decent morphological, conductive, and charge storage capabilities^15,16^ and has excellent conductivity, open porous nanosheet, and flexible oxidation states, thereby enhancing its suitability for use in supercapacitors.^17–19^ Pristine MXene shows excellent supercapacitor performance, but it still has some problems, i.e., restacking of MXene nanosheets.^20^ Hence, 2D MXene has decreased capacitance and stability, is a nonreactive nanosheet, and is restricted to electrolyte diffusion into the interior part of MXene.^21^ There is a need to enhance the supercapacitor performance, such as supercapacitor energy density, rate performance and stability.^22–24^ Among various transition-metal oxide materials, Bi_2_O_3_ has been considered one of the excellent electrodes in supercapacitors due to its low cost, numerous redox activities, extensive operating potential of about 1 V, abundant availability, high-surface area, and eco-friendliness.^25,26^ Bi_2_O_3_ can exhibit a theoretical capacitance around 1370 F g^–1^ and low electrochemical conductivity, which is responsible for increased electrolyte ions transfer in between the electrode and electrolyte interface.^27,28^ To overcome this limitation, significant research efforts are concentrated on Bi_2_O_3_-based nanocomposite electrodes for improving the supercapacitor performance, concerned in terms of energy density, owing to its wide potential window of about 1 V. Importantly, integrating both Bi_2_O_3_ and MXene is a negative electrode material, with a superior supercapacitor performance. Decorating MXene with Bi_2_O_3_ nanoflowers eventually improved the electrical conductivity and electrochemical performance.^29,30^ The presence of the plenty functional group over the MXene nanosheet surface is responsible for assembling the Bi_2_O_3_ electrode via electrostatic interaction. Thus, there is an interest to integrate Bi_2_O_3_ and MXene to a functional composite. Although the synthesis of MXene/Bi_2_O_3_ nanocomposites for supercapacitors has been previously reported, obtaining uniform decoration of Bi_2_O_3_ on MXene nanosheets is a significant challenge. Additionally, a real-world prototype energy storage device based on MXene/Bi_2_O_3_ nanocomposites is still rare. Hence, the advanced progress in MXene/Bi_2_O_3_ electrodes with higher electrochemical performance is an essential and promising direction for energy storage devices. Therefore, it is imperative to develop systematic and rational techniques to construct well-defined MXene/Bi_2_O_3_ electrodes for supercapacitors.

This work presented an MXene/Bi_2_O_3_ nanocomposite supercapacitor electrode on a stainless-steel mesh by using a straightforward, simple coprecipitation method. The electrochemical analysis exhibited a specific capacitance of 613 F g^–1^ and 87% stability over 5000 cycles. The assembled pouch-type MXene/Bi_2_O_3_//MnCo_2_O_3_ asymmetric supercapacitor exhibits higher energy and power performance. Thus, the room-temperature coprecipitation synthesis strategy offers a breakthrough pathway for synthesizing outstanding MXene-based nanocomposite electrodes with excellent energy output and an ideal energy solution for portable electronics.

## Results and Discussion

The assembly of Bi_2_O_3_ nanoflowers over MXene nanosheets was obtained through a simple and easy coprecipitation method and fabricated a pouch-type supercapacitor device, configured as MXene/Bi_2_O_3_//MnCo_2_O_4_. MXene was obtained from the MAX phase via a subsequent exfoliation. After the formation of the MXene phase, Bi_2_O_3_ nanoflower was deposited over the MXene nanosheets, wherein homogeneous precursor solution of bismuth nitrate pentahydrate solution was mixed into a well-dispersed MXene suspension.

This self-assembled process was made possible due to the available surface functional groups of –OH, –O, and –F on the MXene surface, which acts as active sites for assembling oppositely charged Bi^+^ onto the MXene surface via strong chemical attraction through van der Waals interaction,^29^ resulting in a randomly distributed Bi_2_O_3_ nanoflower over the MXene nanosheets (as detailed in the experimental procedure in the Experimental Section) and the corresponding sample was labeled as MXene/Bi_2_O_3_. For comparison purposes, the pristine Bi_2_O_3_ electrode was prepared without adding MXene, and the SEM images of the pristine 2D MXene nanosheet and Bi_2_O_3_ nanoflower have been added in the Supporting Information in Figures S1 and S2. An SEM image of MXene/Bi_2_O_3_ (see Figure 1A) shows a randomly distributed Bi_2_O_3_ nanoflower embedding into a 2D MXene nanosheet, and with closer inspection, it was observed that the prepared Bi_2_O_3_ nanoflower was formed of thin nanoplates and arranged as curled shaped, as result porous structure was formed, which may offer a more efficient electrolyte ion transport path which is essential for electrochemical applications.

**Figure 1:**
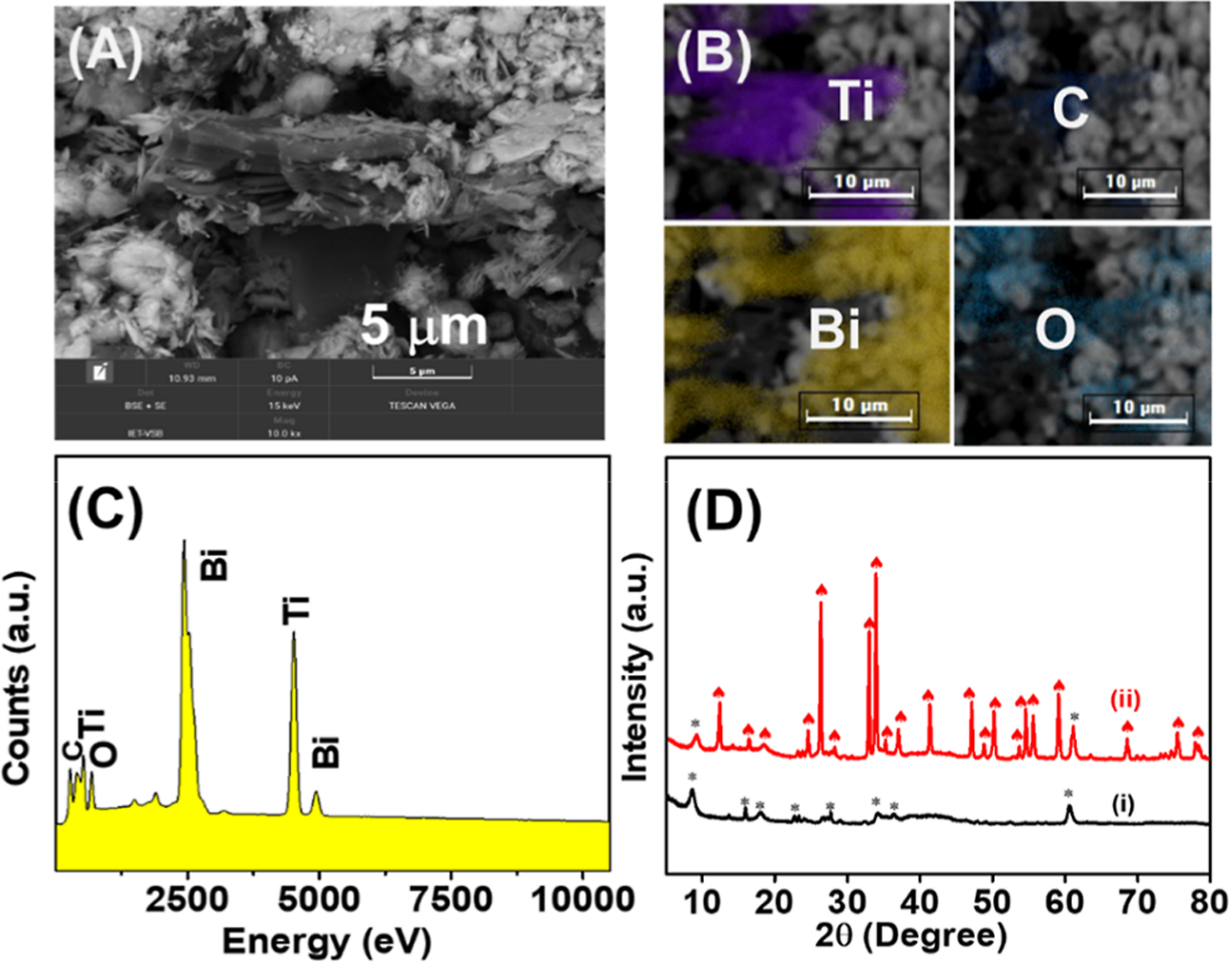
Morphological and structural characterization of MXene/Bi_2_O_3_ nanocomposites: (A) SEM image; (B,C) chemical composition including Ti, Bi, C, and O elements mapping and EDX spectra of MXene/Bi_2_O_3_ nanocomposites; (D) XRD pattern of (i) MXene and (ii) MXene/Bi_2_O_3_ nanocomposites.

In Figure 1B, SEM–EDX color images of the MXene/Bi_2_O_3_ nanocomposite exhibit the uniform distribution of Ti, Bi, C, and O, respectively. The SEM–EDX result proves the effective synthesis of the MXene/Bi_2_O_3_ nanocomposite. The EDX spectra (Figure 1C) reveal that the existence of reflected peaks of Ti, C, Bi, and O elements was corroborative of the successfully prepared MXene/Bi_2_O_3_ nanocomposite at the room-temperature coprecipitation method. Figure 1D­(i,ii) illustrates the XRD patterns of MXene and MXene/Bi_2_O_3_ nanocomposites. The peaks (marked as “*”) displayed at ∼8.48°, ∼18.59°, ∼34.0°, ∼36.52°, and ∼60.76° correspond to the of MXene. Additionally, the lower intensity peaks (marked as “*”) were reflected at ∼15.86°, ∼23.52°, and ∼27.74° related to the existence of TiOF_2_ and TiO_2_.^17,31–33^ However, in the XRD patterns (Figure 1D­(ii)) of MXene/Bi_2_O_3_, peaks, marked as “
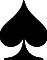
”, reflected at the 2-theta position as ∼12.46°, ∼16.57°, ∼18.75°, ∼24.63°, ∼26.29°, ∼28.18°, ∼32.96°, ∼34.19°, ∼35.28°, ∼37.09°, ∼41.59°, ∼47.24°, ∼48.84°, ∼50.28°, ∼53.84°, ∼54.57°, ∼55.72°, ∼59.21°, ∼68.84°, ∼75.50°, and ∼78.56° related to Bi_2_O_3_ (JCPDS: 96-101-0005). In addition, the MXene peaks were reflected with lower intensity peaks located at ∼8.48° and ∼60.76°, implying that the Bi_2_O_3_ nanoflowers were designed on the MXene nanosheets. In contrast, the XRD pattern of the MXene/Bi_2_O_3_ nanocomposite exhibited a clear shift in the (002) toward the higher angle side, corresponding to an increase in interlayer spacing in MXene layers. This implies that the decorated Bi_2_O_3_ nanoflowers can competently enlarge the interlayer spacing of MXene, which smoothens the electrolyte ions’ diffusion during the adsorption/desorption process. The XRD result proved that the synthesis of MXene/Bi_2_O_3_ nanocomposites was achieved at the room-temperature coprecipitation method; overall, the XRD result is very well aligned with the surface morphology result.

The three electrochemical analyses using cyclic voltammogram, charge–discharge, and electrochemical impedance spectroscopy (EIS) of MXene/Bi_2_O_3_ nanocomposites, MXene, and Bi_2_O_3_ electrodes were carried out in a 6 M KOH electrolyte. Figure 2A–C exhibits the cyclic voltammogram of MXene/Bi_2_O_3_ nanocomposites, Bi_2_O_3_, and MXene were analyzed within the voltage range (scan rate) −1 to 0 V (1–10 mV s^–1^). The cyclic voltammogram of MXene/Bi_2_O_3_ nanocomposite, Bi_2_O_3,_ and MXene shows an almost symmetric nature at different scan rates, revealing excellent reversibility.^25–28^ In Figure 2A–C, with a higher scan rate, the current response of the prepared electrode was increased systematically without notable alteration in its profile of the cyclic voltammogram. As compared to the cyclic voltammogram (see Figure 2D), the (iii) MXene/Bi_2_O_3_ nanocomposite displays a higher current and area under the curves because of the combined consequence of Figure 2D (ii) Bi_2_O_3_ and (i) MXene.

**Figure 2:**
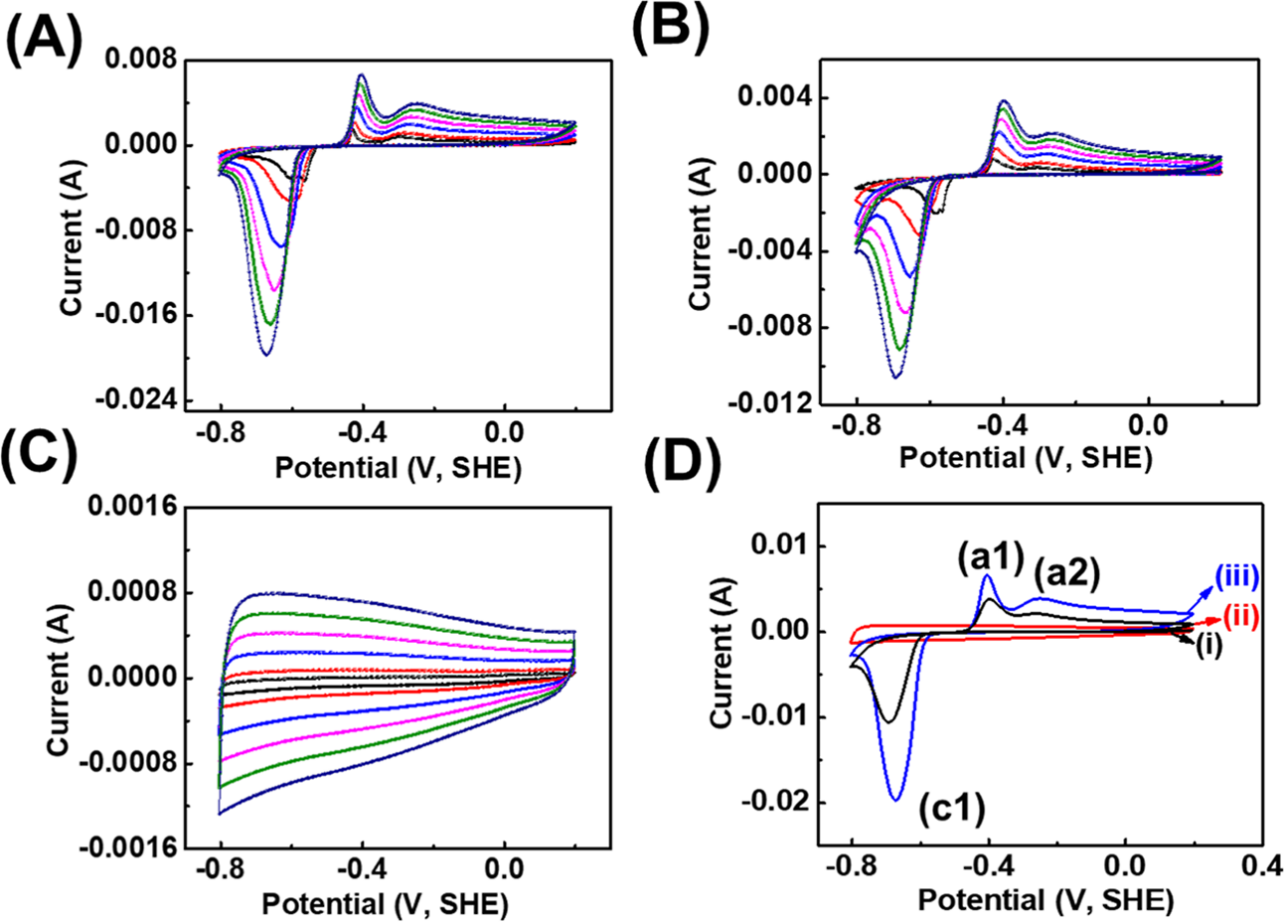
Electrochemical performance analysis: cyclic voltammogram of (A) MXene/Bi_2_O_3_, (B) Bi_2_O_3_, and (C) MXene electrodes at different scan rates (1–10 mV s^–1^) within the potential window 0 to −1 vs SHE, using a 6 M KOH electrolyte; (D) comparative cyclic voltammogram of (i) MXene, (ii) Bi_2_O_3_, and (iii) MXene/Bi_2_O_3_ electrodes at a fixed scan rate of 10 mV s^–1^; (D) in the comparative cyclic voltammogram, the peaks a1, a2, and c1 correspond to the oxidation and reduction processes of the (ii) Bi_2_O_3_ and (iii) MXene/Bi_2_O_3_ electrodes, respectively, confirming their electrochemical activity.

As seen from Figure 2D, there are reflected cathodic and anodic peaks, the single main cathodic peak (marked as c1) reflected at–0.67 (±0.01). However, a pair of anodic peaks (“a1” and “a2”), reflected at–0.39 (±0.01) and–0.24 (±0.01) V, corresponded to a change in its oxidation state from Bi (metal) to Bi­(III). Further, the prolonged voltage plateau is nearly about −0.3 (±0.025), with two oxidation peaks corresponding to BiOOH/Bi­(OH)_3._ ^25–30^

The cathodic peak is displayed at position “c1”, assigned to the following redox reaction

1 $$2H_{2}O+{3{BiO}_{2}}^{2-}\to {{2BiO}_{2}}^{-}+4{OH}^{-}+{Bi}^{\left(0\right)}$$

2 $${Bi}^{\left(0\right)}\to Bi\left(metal\right)$$

Oxidation peaks at “a1” and “a2”, with a voltage plateau region and whose redox reaction is assigned as below

3 $$Bi\left(metal\right)\to {Bi}^{+}+e^{-}$$

4 $${3Bi}^{+}\to {Bi}^{3+}+2Bi\left(metal\right)$$

5 $${3OH}^{-}+{Bi}^{3+}\to {Bi\left(OH\right)}_{3}$$

6 $${Bi\left(OH\right)}_{3}\to BiOOH+H_{2}O$$

Figure 3A–C displays the current-controlled charge–discharge curves of MXene/Bi_2_O_3_ nanocomposites, Bi_2_O_3_, and MXene electrode under varying current densities as (a) 0.8 A g^–1^, (b) 0.7 A g^–1^, (c) 0.6 A g^–1^ , and (a) 3 A g^–1^, (b) 2 A g^–1^, (c) 1 A g^–1^, respectively. As seen from the charge–discharge curves of MXene/Bi_2_O_3_ and Bi_2_O_3_, a nonsymmetrical nature was observed. The discharge curve has been divided into two parts: internal resistance drops and voltage plateaus due to the quasi-Faradaic process.^25–27^ The charge–discharge curves of pristine MXene also exhibit slight nonsymmetry due to its pseudocapacitive mechanisms. In the evaluation of charge–discharge curves (see Figure 3D­(i–iii)) of Bi_2_O_3_, MXene, and MXene/Bi_2_O_3_ electrodes, the prepared MXene/Bi_2_O_3_ nanocomposites have higher charge–discharge times as compared to pristine MXene and Bi_2_O_3_. The specific capacitance values of MXene/Bi_2_O_3_ nanocomposites, Bi_2_O_3_, and MXene electrodes were obtained approximately 613, 405, 297 F g^–1^; 263, 246, 232 F g^–1^; and 73, 68, 63 F g^–1^ at applied current density 0.6–0.8 A g^–1^ and 1–3 A g^–1^, respectively (refer to in the Supporting Information for calculations). As obtained value of specific capacitance, the electrochemical performance of MXene/Bi_2_O_3_ nanocomposites was higher than previously reported MXene-based electrode for supercapacitors, for more details see Table S1,^33–42^ which can be determined that the decorated Bi_2_O_3_ over MXene has a substantial upgrading on the electrochemical properties of nanocomposite electrode, which exceeds that of the unadulterated MXene. The lowering of the electrochemical response with increased applied current density is because of the restriction of the electrolyte ions into the MXene/Bi_2_O_3_ nanocomposite. Further, nanoflower Bi_2_O_3_ was wrapped over the MXene surface, improving the electrochemical performance of MXene due to the enhanced conductivity of Bi_2_O_3_. Interestingly, the nanoflower Bi_2_O_3_ covers the MXene surface, not only preventing the restacking and agglomeration of MXene nanosheets but also facilitating fast electrolyte adsorption/desorption processes during the electrochemical process.

**Figure 3:**
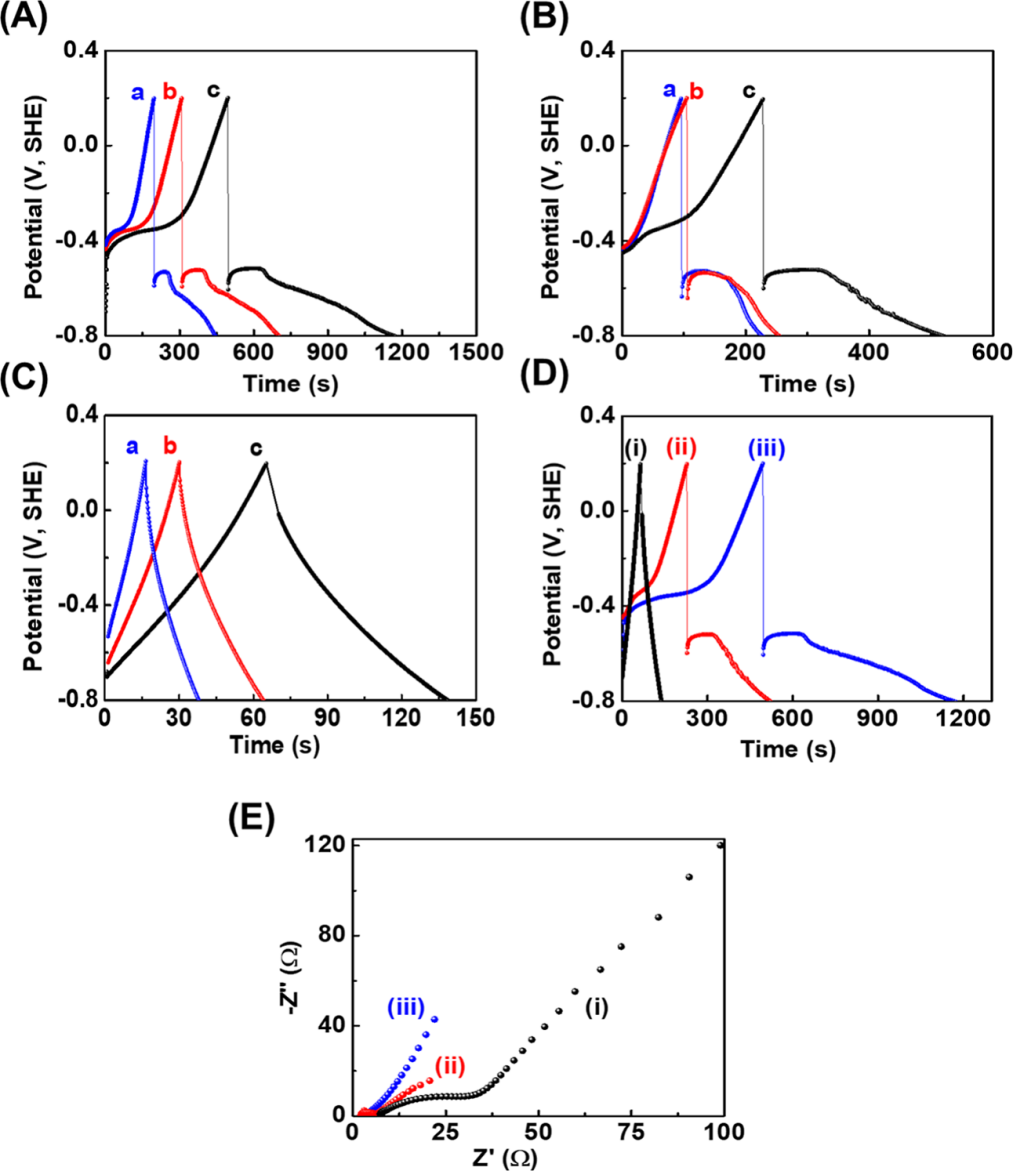
Electrochemical performance analysis: charge–discharge curves of (A) MXene/Bi_2_O_3_, (B) Bi_2_O_3_, and (C) MXene electrodes at various applied current densities as [(a) 0.8 A g^–1^, (b) 0.7 A g^–1^, and (c) 0.6 A g^–1^ for MXene/Bi_2_O_3_, Bi_2_O_3_ electrodes, whereas (a) 3 A g^–1^, (b) 2 A g^–1^, and (c) 1 A g^–1^ for the MXene electrode, respectively]; (D) comparative charge–discharge curves of (i) MXene, (ii) Bi_2_O_3,_ and (iii) MXene/Bi_2_O_3_ electrodes, recorded within the potential window of −1 to 0 vs SHE using a 6 M KOH electrolyte; (E) EIS of (i) MXene, (ii) Bi_2_O_3_, and (iii) MXene/Bi_2_O_3_ electrodes.

Further EIS results for the MXene/Bi_2_O_3_ nanocomposite are shown in Figure 3E. Figure 3E­(i–iii) shows that the charge transfer resistance (*R*_ct_) values of MXene, Bi_2_O_3_, and MXene/Bi_2_O_3_ electrodes were ∼31, ∼6, and ∼4 Ω, respectively. The charge transfer resistance for MXene/Bi_2_O_3_ nanocomposites was lower than that of Bi_2_O_3_ and MXene, indicating that MXene/Bi_2_O_3_ has a more rapid charge transfer rate. Subsequently, with the insertion of nanoflower Bi_2_O_3_ inside and over the MXene surface, the charge transfer resistance of the nanocomposite electrode can be dramatically lowered. This result further reveals that MXene combines with nanoflower Bi_2_O_3_ to construct MXene/Bi_2_O_3_ nanocomposites, improving the conductivity and further enhancing its capacitance.^28–30^ To further investigate the cycling performance of the MXene/Bi_2_O_3_ nanocomposite electrode, a continuous charge/discharge test was performed. The MXene/Bi_2_O_3_ nanocomposite electrode showed higher stability at around 87% over 5000 cycles (Figure S3). The sudden drop in stability after 5000 cycles is due to the dissolution of the active MXene/Bi_2_O_3_ material in an electrolyte solution. Thus, the three-electrode electrochemical result analysis proved that the prepared MXene/Bi_2_O_3_ electrode shows a higher electrochemical performance, which may be a combination of MXene and Bi_2_O_3_ oxide, enhancing the supercapacitor performance. Self-assembled decorated Bi_2_O_3_ over the MXene nanosheet material prevents the restacking issue in the MXene with fast ion transport and efficient absorption and desorption, as well as more active sites, so that the prepared nanocomposite electrode has excellent supercapacitor performance. The overall outstanding electrochemical performance of the MXene/Bi_2_O_3_ nanocomposite electrode was investigated.

To fabricate a laboratory-scale pouch-type MXene/Bi_2_O_3_//MnCo_2_O_4_ electrode, an asymmetric pouch-type supercapacitor device was fabricated using MXene/Bi_2_O_3_ and MnCo_2_O_4_ as a positive and negative electrode (see Scheme 1A,B), respectively. For the fabrication of a pouch asymmetric supercapacitor device, MXene/Bi_2_O_3_ and MnCo_2_O_4_ electrodes were deposited on a stainless-steel mesh as a base substrate (see the experimental procedure in the Experimental section). The morphological, structural, and electrochemical properties of the MnCo_2_O_4_ electrode have been added in Figures S4 and S5. Then, these two electrodes were placed one over the other, and in between them, a paper separator was inserted to prevent short circuits and inserted in a plastic pocket to form a pouch-type asymmetric supercapacitor device (as shown in see Scheme 1C), which contained KOH solution, and then MXene/Bi_2_O_3_//MnCo_2_O_4_ supercapacitor devices were used for powering up portable robots, traffic signal lights, and red/blue LED, respectively.

**Scheme 1:**
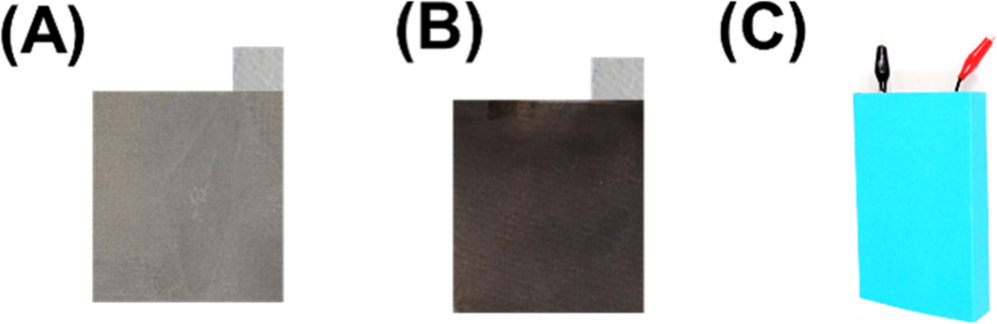
Fabrication of an Asymmetric MXene/Bi_2_O_3_//MnCo_2_O_4_ Supercapacitor for Energy Storage Applications: the Key Steps Involving Fabrication of MXene/Bi_2_O_3_//MnCo_2_O_4_ Asymmetric Devices; (A,B) Digital Photograph of MXene/Bi_2_O_3_ and MnCo_2_O_4_ Electrodes Deposited on Stainless-Steel Mesh Substrates; (C) A Digital Photograph of Laboratory-Scale Fabricated Supercapacitor Devices (i.e., MXene/Bi_2_O_3_//MnCo_2_O_4_)

The cyclic voltammogram of individual MXene/Bi_2_O_3_ and MnCo_2_O_4_ electrodes at a constant scan rate of 10 mV s^–1^ (Figure 4A) indicated that the MXene/Bi_2_O_3_//MnCo_2_O_4_ asymmetric device can be enlarged to 1.45 V, indicating that the higher energy density is directly related to the square of voltage (Figure 4B). The cyclic voltammogram analysis of the MXene/Bi_2_O_3_//MnCo_2_O_4_ device at various scan rates as 5–100 mV s^–1^ displayed an enhanced integrated area with an increased scan rate, signifying an excellent rate capability (Figure 4C).^31^ The charge–discharge curves of the MXene/Bi_2_O_3_//MnCo_2_O_4_ (see Figure 4D) and the profile of the charge–discharge curves were a mirror image of each other, indicating that the supercapacitor device has superior capacitance behavior. Figure 4E shows the Ragone plot of the MXene/Bi_2_O_3_//MnCo_2_O_4_ energy unit, which illustrates the energy and power performance of ∼42 Wh kg^–1^ and 1420 W kg^–1^, respectively. The obtained electrochemical result of the MXene/Bi_2_O_3_//MnCo_2_O_4_ supercapacitor device was outstanding over the previously reported result (Table S2).^33,36,38,41–44^

**Figure 4:**
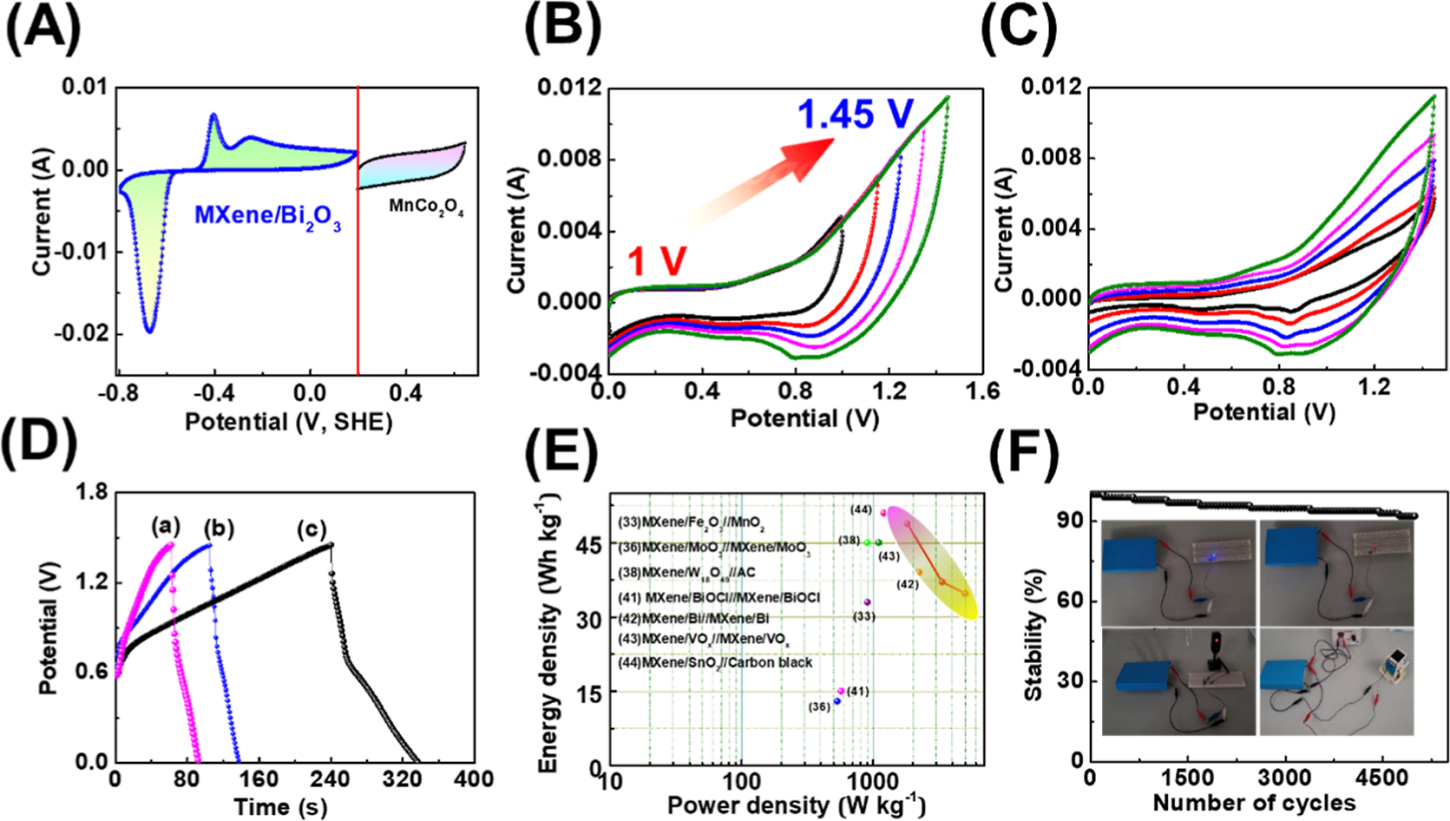
Electrochemical properties of the fabricated MXene/Bi_2_O_3_//MnCo_2_O_4_ asymmetric supercapacitor device: (A) comparison of cyclic voltammogram of MXene/Bi_2_O_3_ and MnCo_2_O_4_ electrodes; (B) extended cyclic voltammogram for different voltages (1–1.45 V) at a fixed scan rate 100 mV s^–1^; (C) cyclic voltammogram at various scan rates (5–100 mV·s^–1^) at a fixed potential range of 0–1.45 V; (D) charge–discharge curves with various current densities as (a) 0.3 A g^–1^, (b) 0.2 A g^–1^, and (c) 0.1 A g^–1^; (E,F) Ragone plots (the inset shows energy and power values of the reported supercapacitor device^33,36,38,41–44^ ) and stability of the MXene/Bi_2_O_3_//MnCo_2_O_4_ (the inset displays digital photographs of unlocking a red/blue LED, a traffic light system, and small portable robots, importance fabricated MXene/Bi_2_O_3_//MnCo_2_O_4_ energy unit for real practical application point of view) (digital photographs taken during powering portable electronic devices by MXene/Bi_2_O_3_//MnCo_2_O_4_ supercapacitors).

The stability of the fabricated MXene/Bi_2_O_3_//MnCo_2_O_4_ supercapacitor device was recorded over 5000 cycles, as shown in Figure 4F, and an obtained stability of around 92%. In the end, the fabricated MXene/Bi_2_O_3_//MnCo_2_O_4_ device was used for practical applications, such as unlocking small portable robots, lighting red/blue LED, and traffic signal lights. For further details, refer to the Supporting Information (Movie S1) provided in the Supporting Information. Therefore, these results are anticipated to open new avenues for the progress of MXene-based nanocomposites for advanced energy storage applications.

## Conclusion

In summary, Bi_2_O_3_ nanoflowers assembled an MXene electrode through a room-temperature coprecipitation method. The synthesis of MXene/Bi_2_O_3_ nanocomposites has revealed a higher electrochemical performance. The pouch-type MXene/Bi_2_O_3_//MnCo_2_O_4_ supercapacitor device revealed an energy and power performance of ∼42 Wh kg^–1^ and 1420 W kg^–1^, 92% over 5000 cycles, implying higher supercapacitor properties. Hence, designing a laboratory-scale pouch-type energy storage device could be a valuable insight in the direction of real, practical application of MXene-based nanocomposite electrodes in energy storage applications.

## Experimental Section

### Synthesis of the MXene/Bi_2_O_3_ MXene Composites

Initially, the stainless-steel mesh was ultrasonically cleaned several times in ethanol and distilled water baths, followed by oven-drying at 50 °C for 12 h. For synthesis, the MXene/Bi_2_O_3_ nanocomposite was deposited over the stainless-steel mesh. In brief, 0.1 M bismuth nitrate pentahydrate was dissolved in 50 mL of double-distilled water; at the later stage, 0.5 mL of 1 M nitric acid and a few drops of liquid ammonia were added to the above solution. Later, the 10 mg of MXene powder was prepared in a solution with continued stirring to form a uniform, homogeneous solution. Finally, 0.5 mL of hydrochloric acid was added, and as a result, precipitation occurred via an oxidation reaction in the reaction precursor solution. After the synthesis of MXene/Bi_2_O_3_ nanocomposites, they were then air-annealed at 400 °C for 3 h. Different analytical methods were employed to analyze the formation of MXene/Bi_2_O_3_ nanocomposites; complete characterization instrumental details have been added in Supporting Information S1.

### Synthesis of the MnCo_2_O_4_ Electrode

The synthesis of the MnCo_2_O_4_ through the hydrothermal method is as follows. 0.05 M cobalt nitrate hexahydrate, manganese nitrate hexahydrate, and 0.1 M urea were mixed in 30 mL of double-distilled water and agitated consistently for 20 min. The prepared solution was loaded into a 50 mL Teflon-lined autoclave; further stainless-steel mesh was kept deep in the prepared solution and sustained at 120 °C for 10 h. Later, the Teflon-lined autoclave was cooled to normal temperature, and well-adherent blackish MnCo_2_O_4_ electrodes were deposited on the stainless-steel mesh substrate. At the end, MnCo_2_O_4_ was heated at 400 °C for 3 h to obtain the MnCo_2_O_4_ electrode.

## Supplementary Material





## Notes

Movie of powering portable electronics using asymmetric supercapacitor devices (MP4)
SEM images of the MXene electrode; SEM images of the Bi_2_O_3_ electrode; stability of MXene/Bi_2_O_3_ nanocomposites over 5000 cycles; material characterizations; morphological study and MXene/Bi_2_O_3_ electrodes; formula; SEM and XRD patterns of the MnCo_2_O_4_ electrode; cyclic voltammogram of MnCo_2_O_4_ at scan rates of 5 mV s^–1^ to 100 mV s^–1^; charge–discharge curves of MnCo_2_O_4_ at current densities of 0.1 to 0.5 A g^–1^; EIS spectrum of the of MnCo_2_O_4_ electrode; half-cell electrochemical performance of MXene/transition-metal oxide-based supercapacitors; and full-cell electrochemical performance of MXene/transition-metal oxide-based super-capacitor devices (PDF)
