# Supplementary material for: MXene/Bi2O3 Nanocomposites as Supercapacitors for Portable Electronic Devices
Source: Energy Fuels. 2025 Oct 27;39(44):21583–9. doi: 10.1021/acs.energyfuels.5c04057 (PMC12598869; doi:10.1021/acs.energyfuels.5c04057)
Supplement: Supplementary file 2 [file ef5c04057_si_002.pdf]

Supporting Information

MXene/Bi_2O_3 Nanocomposites as Supercapacitors for Portable Electronic Devices

Nanasaheb M. Shinde and Martin Pumera*

Advanced Nanorobots & Multiscale Robotics Laboratory, Faculty of Electrical Engineering and Computer Science, VSB - Technical University of Ostrava, 17. listopadu 2172/15, 70800 Ostrava, Czech Republic

* Author for correspondence: M. Pumera, (pumera.research@gmail.com)

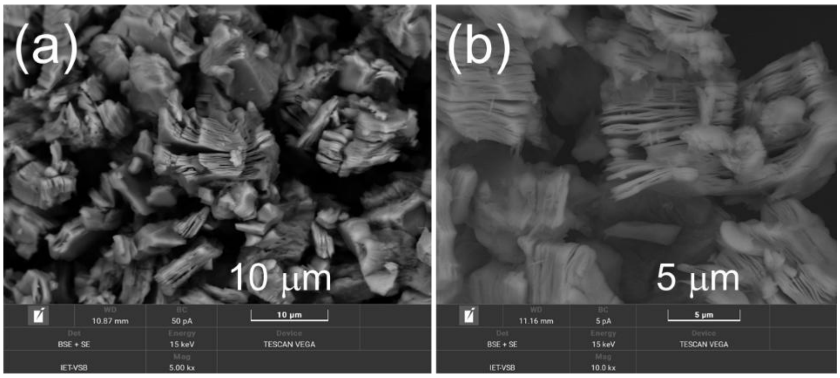

Figure S1. (a and b) SEM images of the MXene electrode.

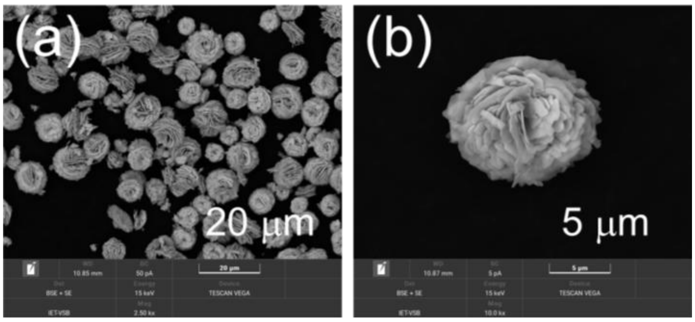

Figure S2. (a and b) SEM images of the Bi_2O_3 electrode.

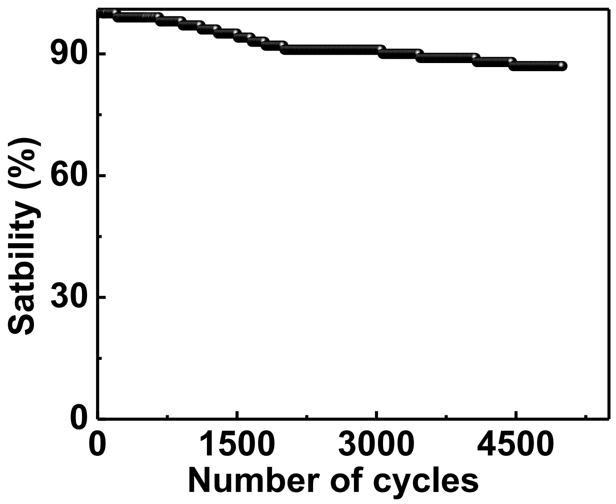

Figure S3. Stability of MXene/Bi_2O_3 nanocomposite over 5000 cycles.

S1. Materials Characterizations: Morphology study and MXene/ Bi_2O_3 electrodes

X-ray diffraction (XRD, Rigaku Smart Lab for Cu–K radiation, =0.1541 nm) was performed to determine the crystallographic information and phase features of the product. For microscopic investigations a scanning electron microscope (SEM: Tescan Vega) with a Tungsten cathode was used. Micrographs were obtained using a combination of secondary electrons (SE) and backscattered electrons (BSE) mode (SE+BSE) and energy-dispersive X-ray spectroscopy (EDS: EDAX) with an acceleration voltage of 15 KeV. The electrochemical characterizations of the manufactured electrodes, including cyclic voltammogram, charge-discharge, and electrochemical impedance spectra (EIS), were investigated using an electrochemical workstation (Metrohm, Netherlands). A frequency range of 0.01 Hz to 100 kHz and a sinusoidal potential of 5 mV were used to study all electrodes with EIS at open circuit potential. charge-discharge profiles were used to determine the stability test from gravimetric capacitance values.

S2. Formulae

The charge-discharge curve-inspired SC of MXene, Bi_2O_3, and MXene/ Bi_2O_3 electrodes were estimated according to the following equation;

$$C = \frac{I\Delta t}{m\Delta V} \tag{1}$$

Where I is the discharge current, Δt, and m is the discharge time and mass of active material, ΔV is the potential window.

Electrochemical quantities like energy density (*ED)* and power density (*PD)* were calculated by the following equations;

Energy density

$$ED = \frac{1}{2} C \Delta V^2 \tag{2}$$

Power density

$$PD = \frac{E}{\Delta t} \times 3600 \tag{3}$$

In this calculation formula, C is the specific capacitance, $\triangle V$ is the potential window (V), $\triangle t$ is the discharging time of the prepared materials.

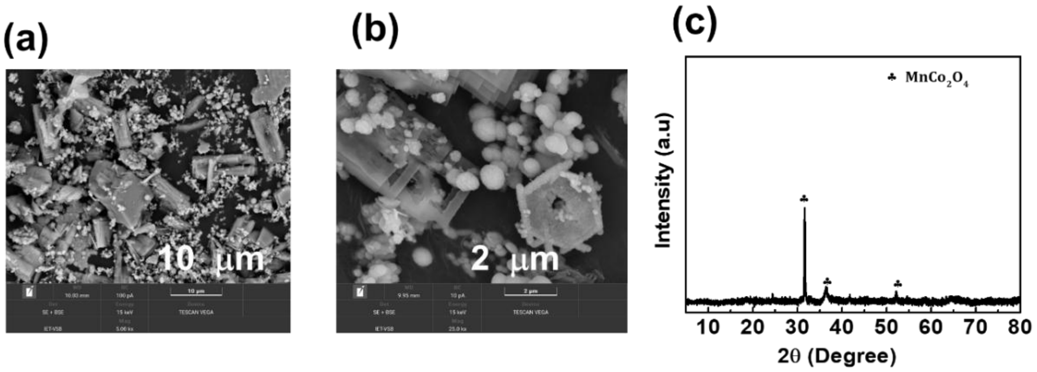

Figure S4. (a and b) SEM and (c) XRD pattern of $MnCo_2O_4$ electrode.

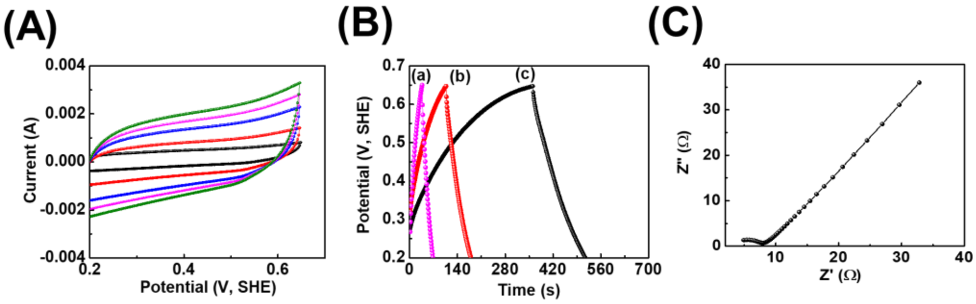

Figure S5. (A) Cyclic voltammogram of $MnCo_2O_4$ at scan rates of 5 mV s^{-1} to 100 mV s^{-1};(B) Charge-discharge curves of $MnCo_2O_4$ at current densities ranging from (a) 0.5 A g^{-1} ; (b) 0.3 A g^{-1}, and (c) 0.1 A g^{-1}; (C) EIS spectrum of $MnCo_2O_4$ electrode.

Table S1. Half-cell Electrochemical performance of MXene/transition metal oxides-based supercapacitors

Sr. No.	Material	Synthesis Method	Specific Capacitance ($F.g^{-1}$)	Stability/Cycles	Ref.
1	MXene/Fe_2O_3	Hydrothermal	486 F g^{-1}	95%/5000	[33]
2	MXene/α-Fe_2O_3/C	Hydrothermal	40 F g^{-1}	-----	[34]
3	MXene/α-Fe_2O_3	Electrostatic	197 F g^{-1}	97%/2,000	[35]
4	MXene/MoO_3	Vacuum filtration	545 F g^{-1}	100%/5,000	[36]
5	MXene/MoO_3	Solvothermal	1680 F g^{-1}	70%/1,000	[37]
6	MXene/$W_{18}O_{49}$	Hydrothermal	500 F g^{-1}	85%/10,000	[38]
7	MXene/RuO_2	Chemical solution	612 F g^{-1}	97%/10,000	[39]
8	MXene/RuO_2	Hydrothermal	388 F g^{-1}	80%/10,000	[40]
9	MXene/BiOCl	Chemical bath deposition route	396.5 F cm^{-3}	80%/10,000	[41]
10	MXene/Bi	Solid-state method	155 F g^{-1}	87%/5000	[42]
**11**	**MXene/Bi_2O_3**	**Coprecipitation**	**613 F g^{-1}**	**87% /5000**	**Current work**

Table S2. Full-cell Electrochemical performance of MXene/transition metal oxides-based supercapacitor device

**Sr. No**	**supercapacitor device**	**ED ($Wh. Kg^{-1}$)**	**PD ($W. Kg^{-1}$)**	**Stability/cycles**	**Ref**
1	MXene/Fe_2O_3// MnO_2	33	900	95%/5000	[33]
2	MXene/MoO_3// MXene/MoO_3	13	534	93%/5000	[36]
3	MXene/$W_{18}O_{49}$//AC	45	900	85%/10000	[38]
4	MXene/BiOCl// MXene/BiOCl	15	570	85%/5000	[41]
5	MXene/ Bi// MXene/Bi	39	2225	93%/3000	[42]
6	MXene/VO_x// MXene/VO_x	45	1100	78/5000	[43]
7	SnO_2/MXene//Carbon black	51	1200	101/10000	[44]
**8**	**MXene/Bi_2O_3//$MnCo_2O_4$**	**42**	**1420**	**92%/5000**	**Current work**
